# Examining the effects of race/ethnicity and other factors on outcomes of care for complex regional pain syndrome type 1 in the United States

**DOI:** 10.1371/journal.pgph.0004022

**Published:** 2025-01-08

**Authors:** Anh Khoa Vo, James A. G. Crispo, Lisa J. W. Liu, Dylan Thibault, Allison W. Willis, Donald E. G. Griesdale, John L. K. Kramer, Jacquelyn J. Cragg

**Affiliations:** 1 Collaboration for Outcomes Research and Evaluation (CORE), Faculty of Pharmaceutical Sciences, University of British Columbia, Vancouver, British Columbia, Canada; 2 International Collaboration on Repair Discoveries (ICORD), University of British Columbia, Vancouver, British Columbia, Canada; 3 Human Sciences Division, NOSM University, Sudbury, Ontario, Canada; 4 Department of Neurology, University of Pennsylvania Perelman School of Medicine, Philadelphia, Pennsylvania, United States of America; 5 Department of Biostatistics, Epidemiology and Informatics, University of Pennsylvania Perelman School of Medicine, Philadelphia, Pennsylvania, United States of America; 6 Department of Anesthesiology, Pharmacology & Therapeutics, Faculty of Medicine, University of British Columbia, Vancouver, British Columbia, Canada; 7 Centre for Clinical Epidemiology and Evaluation, Vancouver Coastal Health Research Institute, Vancouver, British Columbia, Canada; University of Embu, KENYA

## Abstract

Complex regional pain syndrome is a chronic pain disorder marked by symptoms such as swelling, impaired motor function, and sympathetic dysfunction. Our primary objective was to determine the total number of complex regional pain syndrome type 1 (CRPS-1) emergency department (ED) visits and hospitalizations by race/ethnicity, as well as to assess sex and age distributions by race/ethnicity. Secondary objectives were to examine whether race/ethnicity, as well as select characteristics, are associated with hospitalization and longer length of stay. We completed a cross-sectional study of adults (19+ years) using acute and inpatient care data from the 2020 Nationwide Emergency Department Sample and the National Inpatient Sample. The overall rate of CRPS-1 diagnosis among ED visits and hospitalizations was 0.02% and 0.04%, respectively. Most CRPS-1 care was provided to White (ED: 83.1%; inpatient: 82.8%) patients. Within race/ethnicity groups, CRPS-1 ED visits and hospitalizations generally increased with age. Secondary findings included: 1) ED visits by Black individuals (compared with White) were significantly negatively associated with immediate hospitalization (adjusted odds ratio (AOR) 0.74, 95% CI 0.55 to 0.99); 2) hospitalizations by Black patients (compared with White) were independently associated with increased length of stay (odds ratio (OR) 1.45, 95% CI 1.07 to 1.96), though the association diminished with adjustment; and 3) drug abuse was significantly associated with hospitalization (AOR 4.67, 95% CI 3.53 to 6.18) and longer length of stay (AOR 1.81, 95% CI 1.34 to 2.46). Race/ethnicity was minimally associated with studied CRPS-1 outcomes. Additional studies are required to determine the impact of race/ethnicity on seeking care for CRPS-1.

## 1. Introduction

Complex regional pain syndrome (CRPS) is a chronic pain disorder that is divided into two distinct clinical subtypes, CRPS-1 and CRPS-2 [1]. The vast majority of cases belong to CRPS-1, which occurs after an injury, and in contrast to CRPS-2, does not involve direct nerve injury [2,3]. Marked by symptoms such as swelling, impaired motor function, and sympathetic dysfunction [4,5], CRPS-1 poses a diagnostic challenge due to its complex etiology and variable clinical manifestations [1].

One study in the United States (US) found an incidence rate of rate of 5.46 per 100,000 person years at risk between 1989 and 1999 [6]. Another larger US study reported that a diagnosis of CRPS-1 was made during approximately 0.07% of all hospitalizations, including elective admissions, between 2007 and 2011 [7]. While prompt treatment may lead to a better prognosis in CRPS [8], the economic burden is very high, with one study showing that the median total cumulative healthcare cost (inpatient and outpatient) eight years after diagnosis was $43,026, with another $12,037 attributed to pain prescriptions alone [9].

A number of factors have been reported to be associated with the diagnosis of CRPS-1, including trauma to upper extremities, and certain sociodemographic characteristics, such as older age and female sex [7,10,11]. Additionally, racial and ethnic disparities in the diagnosis of CRPS-1 have been observed, whereby, compared with patients of non-White race/ethnicity, White patients experienced a higher rate of CRPS-1 diagnosis after adjusting for other factors [7,12].

Although studies have reported significant race/ethnic differences in the diagnosis of CRPS-1, little is known about whether similar differences exist in care for CRPS-1. Prior studies have highlighted differences in the diagnosis and management of other health conditions, as well as associated health outcomes, by race/ethnicity [13,14]. For example, Morden et al. found that White patients consistently received a higher mean annual dose of prescribed opioids compared with Black patients [13], whereas a separate US study found that racialized groups (including Black, Hispanic, Asian/Pacific Islander, and Native American/other patients) diagnosed with mental health disordeemers experienced a longer inpatient length of stay (LOS) compared with White patients [14]. While there is a general understanding of factors related to CRPS-1 diagnosis, there is a paucity of information on potential disparities in CRPS-1 treatment and outcomes, such as differences, if any, in hospital admission and length of stay by race/ethnicity.

To address knowledge gaps related to potential race/ethnicity-based disparities in CRPS-1, we analyzed acute care and inpatient administrative data from the year 2020. Our primary objective was to determine the total number of CRPS-1 emergency department (ED) visits and hospitalizations by race/ethnicity, as well as to assess the distribution of sex and age within race/ethnicity groups for each clinical setting. Our secondary objectives were to examine whether race/ethnicity, as well as other individual and hospital characteristics, are associated with immediate hospital admission and longer length of stay for CRPS-1 ED visits and hospitalizations, respectively. Findings from the current study provide nationally representative baseline CRPS-1 epidemiological data upon which effective diagnostic and treatment strategies may be based.

## 2. Methods

### 2.1 Ethics and reporting

Our study utilized publicly available data that did not include any direct patient identifiers. It was therefore exempt from ethics board review by the Office of Research Ethics at the University of British Columbia. Our research was conducted in accordance with terms outlined in the US Agency for Healthcare Research and Quality (AHRQ) Healthcare Cost and Utilization Project (HCUP) Data Use Agreement, which required suppressing small cell counts less than or equal to ten. Study particulars were summarized using the Reporting of studies Conducted using Observational Routinely collected Data (RECORD) statement [15].

### 2.2 Data sources and study design

We completed a cross-sectional study within acute and inpatient care settings using 2020 HCUP observational data from the Nationwide Emergency Department Sample (NEDS) and the National (formerly Nationwide) Inpatient Sample (NIS).

The NEDS is the largest all-payer database of ED encounters from American Hospital Association community, non-rehabilitation, hospital-owned EDs in the US [16]. Annual NEDS datasets are constructed from a 20% stratified sample of eligible EDs in a given year, with weights being derived for both individual facilities and visits. When weighted, the more than 28 million distinct ED visits in 2020 represent the roughly 123 million ED encounters in the US for that year. The NIS is the largest publicly available all-payer inpatient care database in the US, and is available yearly dating back to 1988. Starting in 2012, NIS data originate from an approximate 20% sample of discharges from all HCUP-participating hospitals. Derived survey weights may subsequently be used to compute nationally representative estimates of hospital admission and associated outcomes for each year. For 2020, the NIS includes health information from more than seven million distinct hospitalizations. When weighted, these represent more than 35 million unique hospital admissions and account for more than 97% of the country’s population.

The 2020 NEDS and NIS datasets contain in-depth clinical and nonclinical data, such as International Classification of Diseases (Tenth Revision; ICD-10) diagnosis, procedure, and external cause of morbidity codes; patient demographic details (such as age, sex, race/ethnicity, and quartile of median household income); hospital characteristics (such as region, teaching status, trauma center designation, and ownership); and information about healthcare charges and the payer (such as Medicare, private insurance, or no charge). Race/ethnicity is coded as a single variable (*RACE*) within the NEDS and NIS despite the possibility of some HCUP Partner organizations providing separate source data for both race and ethnicity to HCUP. Ethnicity takes precedence when HCUP creates the uniform race values [17].

### 2.3 CRPS-1 encounters

Eligible complex regional pain syndrome type 1 (CRPS-1) ED visits for our study included all ED discharges between January 1, 2020 and December 31, 2020 where an ICD-10-CM diagnosis code for CRPS-1 was recorded in the NEDS as either a primary (DX1) or secondary (DX2-35) diagnosis: CRPS-1, unspecified (G90.50), CRPS-1 of upper limb (G90.51), CRPS-1of lower limb (G90.52), or CRPS-1 of other specified site (G90.59). Pediatric (<19 years) CRPS-1 ED visits were then excluded so that our findings may be compared with the largest epidemiological study of CRPS-1 in the US [7]. We further excluded ED visits where patient race/ethnicity was missing to facilitate the reporting of patient race/ethnicity by age group.

Similar to the selection of CRPS-1 ED visits, eligible CRPS-1 hospitalizations included all adult inpatient discharges between January 1, 2020 and December 31, 2020 where an ICD-10-CM diagnosis code for CRPS-1 was recorded in the NIS as a primary (DX1) or secondary (DX2-40) diagnosis. We subsequently excluded all elective hospitalizations, as well as hospitalizations with a missing elective/non-elective status, to focus on outcomes of non-elective CRPS-1 care. Hospitalizations were further excluded if patient race/ethnicity was missing.

Lastly, eligible CRPS-1 ED visits and hospitalizations were excluded if a patient’s median household income or primary payer data was missing to permit complete cases analyses, as these factors were presumed to confound examined exposure-outcome relationships.

### 2.4 Study outcomes

Our primary study outcomes were the total number of nationwide ED visits and national hospitalizations for CRPS-1, respectively, by race/ethnicity group, as well as the distribution of sex and age within each race/ethnicity group. Secondary outcomes included immediate hospital admission for CRPS-1 ED visits and longer length of inpatient stay for CRPS-1 hospitalizations. Immediate hospital admissions included both admissions to the same hospital and transfers to other short-term hospitals; transfers to short-term hospitals were presumed to result in an inpatient stay. Inpatients stays greater than three days were defined as longer lengths of stay based on the distribution of time to discharge data and three days being the approximate midpoint of CRPS-1 hospitalizations.

### 2.5 Primary analyses

We estimated the total number of CRPS-1 ED visits and hospitalizations, as well as the number of CRPS-1 encounters in each clinical setting by race/ethnicity (White, Black, Hispanic, Other), using HCUP discharge weights for the 2020 NEDS and NIS, respectively. The distribution of sex (female, male) and age (19–29, 30–39, 40–49, 50–59, 60–69, and 70+ years) within CRPS-1 encounters for each setting were then examined and reported as percentages of total CRPS-1 encounters within each race/ethnicity group.

### 2.6 Secondary analyses

Sociodemographic, clinical, and hospital characteristics of study encounters where a diagnosis of CRPS-1 was recorded were summarized using descriptive statistics, as were hospital admission and longer length of stay outcomes related to CRPS-1 ED visits and hospitalizations, respectively. Chi-square tests were used to assess whether the distribution of examined sociodemographic, clinical, and hospital variable values differed by encounter outcome. We used the HCUP Elixhauser Comorbidity Software Refined (version 2023.1) for ICD-10-CM to measure twenty distinct pre-existing medical conditions that did not require a present-on-admission indicator and were assumed to not arise from hospital care (such as metastatic cancer, dementia, and obesity) [18].

We used unconditional logistic regression to determine whether race/ethnicity, as well as other individual and hospital characteristics, were associated with examined secondary outcomes. For these analyses, we constructed the following four sets of models to measure exposure-outcome associations for each secondary outcome: 1) *bivariable models* for each reported characteristic; 2) a *base adjusted model*, which included race/ethnicity, age, sex, median household income, and primary payer; 3) an *intermediate adjusted model*, which included all *base adjusted model* covariates and comorbidities identified as having a significant association with the study outcome in the *bivariable models*; and 4) a *fully adjusted model*, which included all *intermediate adjusted model* covariates and all other variables where one or more levels were significantly associated with the study outcome in the *bivariable models*.

Covariates included in the *base adjusted model* were selected a priori based on clinical knowledge and their presumed ability to confound examined exposure-outcome relationships. All of our statistical models accounted for the complexity of NEDS and NIS survey designs, including the clustering of discharges within hospitals, to generate precise variance estimates for reported odds ratios. A significance level of 0.05 was used for all analyses.

### 2.7 Software

We used SAS V.9.4 (SAS Institute Inc., Cary, North Carolina, USA) to complete all analyses. GraphPad Prism Version 9.2.0 (GraphPad Software LLC, San Diego, California, USA) was used to graphically report 2020 CRPS-1 counts and related proportions.

## 3. Results

### 3.1 Emergency department visits for CRPS-1

**Fig 1:**
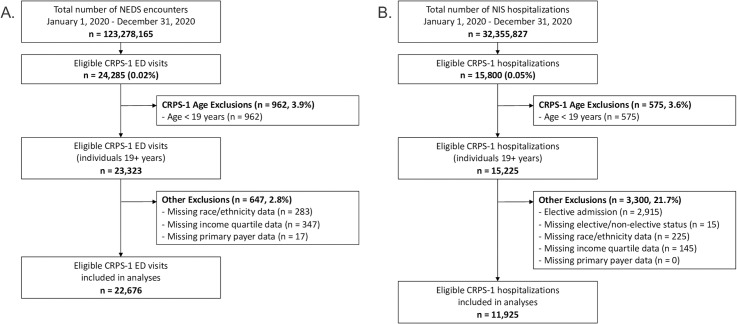
Selection of CRPS-1 emergency department encounters (A) and hospitalizations (B). Abbreviations: CRPS-1, complex regional pain syndrome type 1; ED, Emergency department; NEDS, Nationwide emergency department sample; NIS, National (Nationwide) inpatient sample.

The selection of ED encounters where CRPS-1 was diagnosed is shown in Fig 1A. Between January 1, 2020 and December 31, 2020, there were 23,323 adult encounters where CRPS-1 was diagnosed. After excluding encounters with missing race/ethnicity data (283, 1.2%), a total of 23,040 ED CRPS-1 visits remained. The overall rate of CRPS-1 diagnosis among ED visits was 0.02%; CRPS-1 was documented as a primary diagnosis in 11.0% of ED visits (89.6% secondary diagnosis; 0.6% primary and secondary diagnosis).

**Fig 2:**
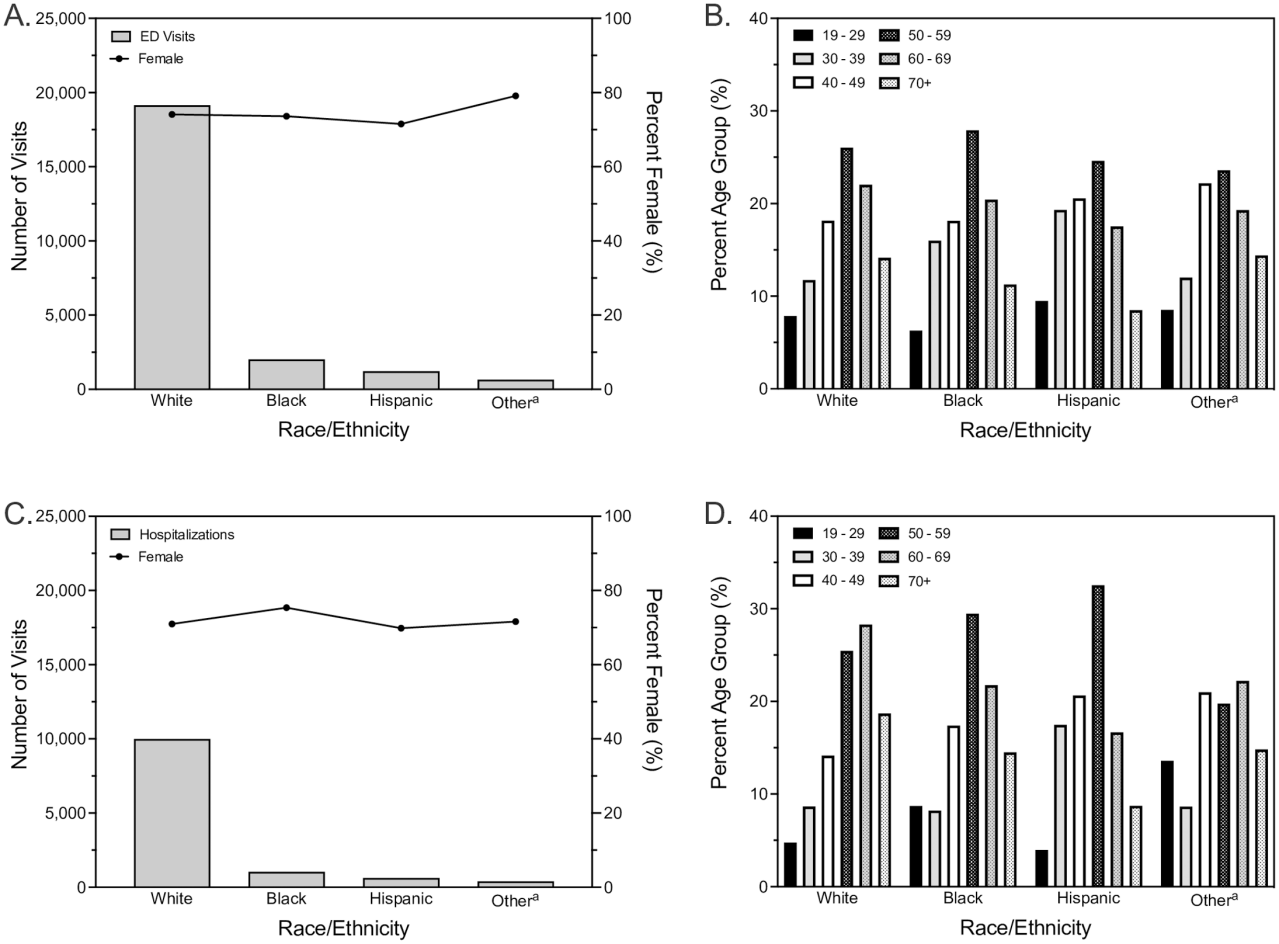
Adult emergency department visits and hospitalizations in 2020 for complex regional pain syndrome type 1 (CRPS-1). Total number of CRPS-1 emergency department visits and proportion female by race/ethnicity (A), and age distribution of CRPS-1 by race/ethnicity (B). Total number of CRPS-1 hospitalizations and proportion female by race/ethnicity (C), and age distribution of CRPS-1 by race/ethnicity (D). Abbreviation: ED, Emergency department. ^a^Includes Asian/Pacific Islander, Native American, two or more races, multiracial, and any race/ethnicity other than White, Black, or Hispanic.

White populations (19,139 visits; 83.1% of total) accounted for the greatest number of annual CRPS-1 ED visits, followed by Black (2,024 visits; 8.8% of total), Hispanic (1,231 visits; 5.3% of total) and other race/ethnicity populations (646 visits; 2.8% of total) (Fig 2A). Overall, female patients visited the ED more often (17,067 visits; 74.1% of total) for CRPS-1 compared with males. There were minor differences in the proportion of ED visits by female patients across examined race/ethnicity groups, with the greatest proportion of females observed among patients of other (79.1%) race/ethnicity (which comprises Asian/Pacific Islander, Native American, two or more races, multiracial, and any race/ethnicity other than White, Black, or Hispanic) and the lowest proportion of females observed among individuals of Hispanic (71.5%) origin (Fig 2A).

Few (1,806 visits; 7.8% of total) CRPS-1 ED visits were by younger adults (19–29 years) in 2020; the highest number of CRPS-1 visits were by individuals 50–59 years of age (6,004 visits; 26.1% of total). Within all distinct race/ethnicity groups, the proportion of CRPS-1 ED visits in each age category increased with increasing age, peaking with those aged 50–59 years, and then steadily declined (Fig 2B). Overall, age distributions in CRPS-1 ED visits were similar across examined race/ethnicity groups.

For our secondary admission analyses (n = 22,676 visits), few ED visits for CRPS-1 from our primary analyses were excluded due to missing income quartile data (347; 1.5% of total) or missing primary payer data (17; 0.1% of total) (Fig 1A). The majority of CRPS-1 ED visits were by patients with Medicare (50.7%) or private insurance (26.0%) and occurred at hospitals in the South (31.3%) and Midwest (26.8%) (Table 1). Most hospitals where ED CRPS-1 care was provided were characterized as: 1) metropolitan teaching (69.5%), 2) privately controlled but not-for-profit (58.7%), and/or 3) non-trauma (52.9%) centres. Comorbidities commonly diagnosed during ED visits included uncomplicated hypertension (34.6%), chronic pulmonary disease (30.3%), depression (22.6%), obesity (18.0%), and hypothyroidism (14.8%).

**Table 1:** Associations between select factors and immediate hospital admission among emergency department encounters where complex regional pain syndrome type 1 was diagnosed, 2020.

Characteristic	All ED Visits n (%)	Admitted to Hospital						
		Yes n (%)	No n (%)					
	n = 22,676	n = 10,868	n = 11,808	p-value^a^	OR	AOR Model 1	AOR Model 2	AOR Model 3
Race/ethnicity								
White	18,871 (83.2)	9,183 (48.7)	9,688 (51.3)	0.1756	Reference	Reference	Reference	Reference
Black	1,959 (8.6)	858 (43.8)	1,102 (56.2)		0.82 (0.64–1.05)	0.87 (0.66–1.14)	0.81 (0.60–1.11)	0.74 (0.55–0.99)*
Hispanic	1,199 (5.3)	495 (41.3)	704 (58.7)		0.74 (0.53–1.04)	0.85 (0.60–1.20)	0.89 (0.61–1.31)	0.87 (0.59–1.27)
Other^b^	646 (2.8)	332 (51.4)	314 (48.6)		1.12 (0.74–1.67)	1.18 (0.77–1.80)	1.31 (0.85–2.04)	1.28 (0.83–1.98)
Age								
19–29	1,792 (7.9)	546 (30.5)	1,246 (69.5)	<.0001	Reference	Reference	Reference	Reference
30–39	2,849 (12.6)	893 (31.4)	1,956 (68.6)		1.04 (0.70–1.55)	1.05 (0.70–1.58)	0.82 (0.55–1.24)	0.82 (0.53–1.25)
40–49	4,177 (18.4)	1,722 (41.2)	2,455 (58.8)		1.60 (1.08–2.38)*	1.58 (1.05–2.40)*	1.15 (0.76–1.73)	1.15 (0.75–1.76)
50–59	5,880 (25.9)	2,818 (47.9)	3,063 (52.1)		2.10 (1.45–3.04)***	2.03 (1.37–2.99)***	1.39 (0.94–2.05)	1.45 (0.98–2.15)
60–69	4,905 (21.6)	2,850 (58.1)	2,054 (41.9)		3.17 (2.15–4.67)***	2.98 (1.95–4.56)***	1.77 (1.16–2.73)**	1.88 (1.20–2.95)**
70–79	2,299 (10.1)	1,516 (65.9)	783 (34.1)		4.42 (2.91–6.71)***	3.90 (2.42–6.30)***	2.14 (1.30–3.52)**	2.28 (1.36–3.84)**
80–90	773 (3.4)	523 (67.6)	250 (32.4)		4.76 (2.79–8.11)***	4.11 (2.29–7.38)***	2.20 (1.13–4.31)*	2.51 (1.27–4.98)**
Sex								
Male	5,877 (25.9)	3,139 (53.4)	2,738 (46.6)	0.0010	Reference	Reference	Reference	Reference
Female	16,799 (74.1)	7,729 (46.0)	9,070 (54.0)		0.74 (0.63–0.88)***	0.73 (0.61–0.86)***	0.71 (0.59–0.85)***	0.70 (0.58–0.85)***
Median household income								
Quartile 4 ($86,000+)	5,673 (25.0)	2,941 (51.8)	2,732 (48.2)	0.2104	Reference	Reference	Reference	Reference
Quartile 3 ($65,000–$85,999)	5,957 (26.3)	2,866 (48.1)	3,091 (51.9)		0.86 (0.68–1.10)	0.91 (0.71–1.17)	0.92 (0.70–1.22)	0.92 (0.70–1.20)
Quartile 2 ($50,000–$64,999)	5,822 (25.7)	2,650 (45.5)	3,172 (54.5)		0.78 (0.61–0.99)*	0.80 (0.62–1.05)	0.83 (0.62–1.11)	0.94 (0.70–1.25)
Quartile 1 ($1–$49,999)	5,224 (23.0)	2,411 (46.2)	2,813 (53.8)		0.80 (0.61–1.04)	0.85 (0.64–1.12)	0.87 (0.63–1.20)	1.05 (0.77–1.43)
Primary payer								
Medicare	11,486 (50.7)	6,277 (54.7)	5,209 (45.3)	<.0001	1.61 (1.34–1.93)***	1.14 (0.93–1.40)	0.94 (0.76–1.16)	0.95 (0.76–1.18)
Medicaid	3,633 (16.0)	1,522 (41.9)	2,110 (58.1)		0.96 (0.75–1.23)	1.08 (0.85–1.38)	0.90 (0.70–1.16)	0.91 (0.70–1.19)
Private insurance	5,900 (26.0)	2,529 (42.9)	3,371 (57.1)		Reference	Reference	Reference	Reference
Self-pay	717 (3.2)	157 (21.9)	559 (78.1)		0.38 (0.23–0.61)***	0.38 (0.24–0.61)***	0.39 (0.25–0.62)***	0.38 (0.24–0.60)***
Other (including no charge)	941 (4.1)	382 (40.6)	559 (59.4)		0.91 (0.63–1.31)	0.89 (0.62–1.28)	0.87 (0.62–1.24)	0.90 (0.62–1.29)
Comorbidities								
Acquired immune deficiency syndrome	94 (0.4)	74 (78.5)	20 (21.5)	0.0282	3.99 (1.25–12.75)*	–	4.20 (0.99–17.75)	4.90 (1.15–20.81)*
Alcohol abuse	645 (2.8)	494 (76.7)	150 (23.3)	<.0001	3.70 (2.47–5.55)***	–	3.67 (2.31–5.84)***	3.35 (2.11–5.32)***
Autoimmune conditions	1,443 (6.4)	937 (64.9)	506 (35.1)	<.0001	2.11 (1.63–2.72)***	–	1.87 (1.36–2.55)***	1.85 (1.35–2.52)***
Lymphoma	76 (0.3)	55 (72.0)	21 (28.0)	0.0365	2.80 (1.02–7.65)*	–	2.54 (0.81–7.97)	2.49 (0.89–6.99)
Leukemia	109 (0.5)	74 (67.6)	35 (32.4)	0.0929	2.28 (0.93–5.58)	–	–	–
Metastatic cancer	239 (1.1)	195 (81.7)	44 (18.3)	<.0001	4.91 (2.30–10.49)***	–	4.80 (2.08–11.06)***	4.36 (1.91–9.96)***
Solid tumor without metastasis, in situ	##	##	##	##	##	–	–	–
Solid tumor without metastasis, malignant	324 (1.4)	218 (67.2)	106 (32.8)	0.0012	2.25 (1.33–3.81)**	–	1.35 (0.72–2.53)	1.31 (0.70–2.46)
Dementia	453 (2.0)	365 (80.5)	88 (19.5)	<.0001	4.61 (2.72–7.80)***	–	2.48 (1.32–4.65)**	2.37 (1.25–4.49)**
Depression	5,114 (22.6)	2,975 (58.2)	2,138 (41.8)	<.0001	1.71 (1.41–2.06)***	–	1.52 (1.24–1.85)***	1.50 (1.23–1.82)***
Diabetes without chronic complications	1,923 (8.5)	893 (46.4)	1,030 (53.6)	0.5807	0.94 (0.74–1.18)	–	–	–
Diabetes with chronic complications	3,301 (14.6)	2,483 (75.2)	818 (24.8)	<.0001	3.98 (3.19–4.96)***	–	2.20 (1.72–2.83)***	2.17 (1.69–2.79)***
Drug abuse	1,908 (8.4)	1,484 (77.8)	424 (22.2)	<.0001	4.25 (3.23–5.60)***	–	4.85 (3.67–6.41)***	4.67 (3.53–6.18)***
Hypertension, complicated	2,831 (12.5)	2,115 (74.7)	716 (25.3)	<.0001	3.74 (2.94–4.77)***	–	1.86 (1.41–2.45)***	1.85 (1.40–2.44)***
Hypertension, uncomplicated	7,852 (34.6)	4,107 (52.3)	3,745 (47.7)	0.0003	1.31 (1.14–1.51)***	–	1.17 (1.01–1.35)*	1.15 (0.99–1.34)
Chronic pulmonary disease	6,882 (30.3)	3,976 (57.8)	2,906 (42.2)	<.0001	1.77 (1.51–2.07)***	–	1.33 (1.12–1.57)**	1.29 (1.09–1.53)**
Obesity	4,072 (18.0)	2,805 (68.9)	1,267 (31.1)	<.0001	2.89 (2.36–3.55)***	–	2.38 (1.91–2.95)***	2.37 (1.91–2.94)***
Peripheral vascular disease	1,277 (5.6)	860 (67.3)	417 (32.7)	<.0001	2.35 (1.62–3.40)***	–	1.41 (0.88–2.25)	1.36 (0.87–2.12)
Hypothyroidism	3,346 (14.8)	2,170 (64.8)	1,176 (35.2)	<.0001	2.26 (1.83–2.77)***	–	1.73 (1.37–2.17)***	1.70 (1.35–2.13)***
Other thyroid disorders	371 (1.6)	227 (61.2)	144 (38.8)	0.0137	1.73 (1.11–2.70)*	–	1.37 (0.84–2.23)	1.36 (0.82–2.26)
Hospital region								
Northeast	5,123 (22.6)	2,685 (52.4)	2,437 (47.6)	0.1580	Reference	–	–	–
Midwest	6,081 (26.8)	2,764 (45.5)	3,317 (54.5)		0.76 (0.53–1.07)	–	–	–
South	7,089 (31.3)	3,501 (49.4)	3,588 (50.6)		0.89 (0.64–1.23)	–	–	–
West	4,383 (19.3)	1,917 (43.7)	2,466 (56.3)		0.71 (0.50–1.00)	–	–	–
Hospital location/teaching status								
Metropolitan non-teaching	4,359 (19.2)	1,952 (44.8)	2,408 (55.2)	<.0001	1.98 (1.44–2.72)***	–	–	1.87 (1.31–2.66)***
Metropolitan teaching	15,761 (69.5)	8,174 (51.9)	7,587 (48.1)		2.63 (1.97–3.52)***	––	–	2.07 (1.49–2.89)***
Non-metropolitan hospital	2,556 (11.3)	742 (29.0)	1,814 (71.0)		Reference	–	–	Reference
Hospital control/ownership								
Government or private (collapsed category)	3,104 (13.7)	1,433 (46.2)	1,670 (53.8)	0.3733	0.91 (0.59–1.42)	–	–	–
Government, non-federal (public)	1,825 (8.0)	757 (41.5)	1,067 (58.5)		0.76 (0.52–1.10)	–	–	–
Private, not-for-profit (voluntary)	13,300 (58.7)	6,467 (48.6)	6,833 (51.4)		1.01 (0.74–1.37)	–	–	–
Private, investor-owned (proprietary)	1,556 (6.9)	754 (48.4)	802 (51.6)		Reference	–	–	–
Private (collapsed category)	2,891 (12.7)	1,456 (50.4)	1,435 (49.6)		1.08 (0.74–1.59)	–	–	–
Hospital trauma level designation								
Not a trauma center	11,996 (52.9)	5,173 (43.1)	6,823 (56.9)	0.0002	0.59 (0.42–0.84)**	–	–	0.61 (0.43–0.87)**
Trauma center level I	3,852 (17.0)	2,165 (56.2)	1,687 (43.8)		Reference	–	–	Reference
Trauma center level II	3,593 (15.8)	2,078 (57.8)	1,515 (42.2)		1.07 (0.70–1.62)	–	–	0.94 (0.63–1.41)
Trauma center level III	3,122 (13.8)	1,406 (45.0)	1,716 (55.0)		0.64 (0.41–0.99)*	–	–	0.60 (0.39–0.94)*
Collapsed category (level I, II, or III)	112 (0.5)	45 (40.0)	67 (60.0)		0.52 (0.20–1.35)	–	–	1.18 (0.73–1.88)

Abbreviations: AOR, adjusted odds ratio; ED, Emergency Department; OR, odds ratio.

^a^Chi-square test.

^b^Includes Asian/Pacific Islander, Native American, two or more races, multiracial, and any race/ethnicity other than White, Black, or Hispanic.

***p < 0.001; **p < 0.01; *p < 0.05.

## Data suppressed; 10 or fewer observations in some cells.

### 3.2 Hospitalizations for CRPS-1

There was a total of 15,225 adult hospitalizations where CRPS-1 was recorded as a diagnosis between January 1, 2020 and December 31, 2020 (Fig 1B). Of these, 2,915 (19.1%) elective admissions were excluded from our analyses, as were 15 (0.1%) and 225 (1.5%) admissions where elective/non-elective status and race/ethnicity data, respectively, was missing. After exclusions, there were 12,070 CRPS-1 hospitalizations included in our primary analyses, corresponding to a CRPS-1 inpatient diagnosis rate of 0.04%. Among eligible hospitalizations, CRPS-1 was documented as a primary diagnosis in 4.6% of admissions (95.9% secondary diagnosis; 0.6% primary and secondary diagnosis).

Overall, patterns of CRPS-1 hospitalizations by race/ethnicity were comparable to our findings from ED settings. White (10,000 admissions; 82.8% of total) and Black (1,035 admissions; 8.6% of total) patients comprised the majority of CRPS-1 hospitalizations, whereas a small number of admissions were by Hispanic (630 admissions; 5.2% of total) and other race/ethnicity patients (405 admissions; 3.3% of total) (Fig 2C). The majority of CRPS-1 hospitalizations were by female patients (8,605 admissions; 71.3% of total), and there were minimal fluctuations in the proportion of hospitalizations by female patients across examined race/ethnicity groups.

As observed for CPRS-1 ED visits, CRPS-1 hospitalizations were infrequent (645; 5.3% of total) among younger adults. The proportion of CRPS-1 hospitalizations in each age category generally increased with increasing age until age 50–59 years for White, Black, and Hispanic populations, though did not peak until age 60–69 years for hospitalizations by White individuals (Fig 2D). Notable differences were observed in the age distribution of CRPS-1 hospitalizations among other race/ethnicity patients, whereby the youngest individuals comprised 13.6% of all admissions.

Few CRPS-1 hospitalizations (145; 1.2% of total) from our primary analyses were excluded for our secondary analyses (n = 11,925 admissions) due to missing income quartile data. No hospitalizations were excluded due to missing primary payer data (Fig 1B). Most CRPS-1 hospitalizations were subsidized by Medicare (56.5%) or private insurance (23.9%), originated in the ED (86.2%), did not include a major operating room procedure (83.7%), and resulted in routine discharge (60.0%) (Table 2). Inpatients stays related to CRPS-1 commonly occurred at hospitals in the South (30.8%) and Midwest (25.1%), as well as centres that were classified as large (49.1%), urban teaching (76.7%), and/or privately controlled but not-for-profit (83.4%). Uncomplicated hypertension (37.4%), chronic pulmonary disease (34.1%), depression (29.0%), obesity (24.7%), and diabetes with chronic complications (20.9%) were the most frequently recorded comorbidities during CRPS-1 hospitalizations.

**Table 2:** Associations between select factors and longer length of stay among inpatient encounters where complex regional pain syndrome type 1 was diagnosed, 2020.

Characteristic		Longer Length of Stay (4+ days)						
	All Admissions n (%)	Yes n (%)	No n (%)					
	n = 11,925	n = 6,605	n = 5,320	p-value^a^	OR	AOR Model 1	AOR Model 2	AOR Model 3
Race/ethnicity								
White	9,900 (83.0)	5,425 (54.8)	4,475 (45.2)	0.0549	Reference	Reference	Reference	Reference
Black	1,020 (8.6)	650 (63.7)	370 (36.3)		1.45 (1.07–1.96)*	1.44 (1.06–1.94)*	1.43 (1.05–1.94)*	1.34 (0.98–1.83)
Hispanic	605 (5.1)	335 (55.4)	270 (44.6)		1.02 (0.70–1.49)	1.06 (0.72–1.56)	1.07 (0.73–1.58)	1.15 (0.76–1.74)
Other^b^	400 (3.4)	195 (48.7)	205 (51.3)		0.79 (0.52–1.19)	0.80 (0.53–1.22)	0.85 (0.55–1.30)	0.84 (0.54–1.31)
Age								
19–29	645 (5.4)	325 (50.4)	320 (49.6)	0.7531	Reference	Reference	Reference	Reference
30–39	1,060 (8.9)	570 (53.8)	490 (46.2)		1.15 (0.73–1.80)	1.10 (0.70–1.73)	1.02 (0.65–1.60)	1.00 (0.63–1.59)
40–49	1,775 (14.9)	1,000 (56.3)	775 (43.7)		1.27 (0.83–1.95)	1.17 (0.76–1.80)	1.08 (0.70–1.66)	1.04 (0.67–1.63)
50–59	3,095 (26.0)	1,690 (54.6)	1,405 (45.4)		1.18 (0.80–1.75)	1.08 (0.72–1.62)	0.99 (0.66–1.47)	0.86 (0.57–1.31)
60–69	3,225 (27.0)	1,780 (55.2)	1,445 (44.8)		1.21 (0.82–1.80)	1.07 (0.71–1.62)	0.95 (0.63–1.44)	0.77 (0.50–1.20)
70–79	1,570 (13.2)	905 (57.6)	665 (42.4)		1.34 (0.87–2.06)	1.15 (0.73–1.82)	0.99 (0.62–1.58)	0.66 (0.41–1.09)
80–90	555 (4.7)	335 (60.4)	220 (39.6)		1.50 (0.89–2.52)	1.27 (0.74–2.18)	1.02 (0.58–1.79)	0.66 (0.36–1.20)
Sex								
Male	3,410 (28.6)	1,870 (54.8)	1,540 (45.2)	0.7384	Reference	Reference	Reference	Reference
Female	8,515 (71.4)	4,735 (55.6)	3,780 (44.4)		1.03 (0.86–1.24)	1.01 (0.84–1.22)	1.04 (0.87–1.26)	0.98 (0.81–1.19)
Median household income								
Quartile 4 ($86,000+)	2,795 (23.4)	1,485 (53.1)	1,310 (46.9)	0.1373	Reference	Reference	Reference	Reference
Quartile 3 ($65,000–$85,999)	3,270 (27.4)	1,760 (53.8)	1,510 (46.2)		1.03 (0.82–1.30)	1.03 (0.82–1.30)	1.02 (0.81–1.30)	1.02 (0.80–1.32)
Quartile 2 ($50,000–$64,999)	2,970 (24.9)	1,635 (55.1)	1,335 (44.9)		1.08 (0.85–1.37)	1.08 (0.85–1.37)	1.06 (0.83–1.36)	1.05 (0.81–1.38)
Quartile 1 ($1–$49,999)	2,890 (24.2)	1,725 (59.7)	1,165 (40.3)		1.31 (1.02–1.68)*	1.27 (0.98–1.63)	1.28 (0.99–1.66)	1.17 (0.88–1.54)
Primary payer								
Medicare	6,735 (56.5)	3,870 (57.5)	2,865 (42.5)	0.0812	1.12 (0.91–1.37)	1.07 (0.86–1.33)	1.00 (0.80–1.24)	0.91 (0.72–1.15)
Medicaid	1,690 (14.2)	820 (48.5)	870 (51.5)		0.78 (0.59–1.03)	0.74 (0.56–0.99)*	0.68 (0.51–0.91)**	0.68 (0.51–0.92)*
Private insurance	2,850 (23.9)	1,560 (54.7)	1,290 (45.3)		Reference	Reference	Reference	Reference
Self-pay	230 (1.9)	125 (54.3)	105 (45.7)		0.98 (0.56–1.73)	0.98 (0.55–1.76)	0.94 (0.54–1.64)	0.85 (0.46–1.56)
Other (including no charge)	420 (3.5)	230 (54.8)	190 (45.2)		1.00 (0.64–1.57)	0.95 (0.61–1.50)	0.92 (0.58–1.45)	0.90 (0.55–1.48)
Comorbidities								
Acquired immune deficiency syndrome	45 (0.4)	20 (44.4)	25 (55.6)	0.5176	0.64 (0.17–2.40)	–	–	–
Alcohol abuse	580 (4.9)	355 (61.2)	225 (38.8)	0.2045	1.29 (0.87–1.90)	–	–	–
Autoimmune conditions	965 (8.1)	545 (56.5)	420 (43.5)	0.7597	1.05 (0.77–1.43)	–	–	–
Lymphoma	75 (0.6)	45 (60.0)	30 (40.0)	0.7147	1.21 (0.43–3.40)	–	–	–
Leukemia	85 (0.7)	30 (35.3)	55 (64.7)	0.1317	0.44 (0.16–1.22)	–	–	–
Metastatic cancer	285 (2.4)	200 (70.2)	85 (29.8)	0.0289	1.92 (1.07–3.46)*	–	2.13 (1.16–3.94)*	1.80 (0.90–3.60)
Solid tumor without metastasis, in situ	##	##	##	##	##	–	–	–
Solid tumor without metastasis, malignant	195 (1.6)	105 (53.8)	90 (46.2)	0.8453	0.94 (0.50–1.77)	–	–	–
Dementia	290 (2.4)	205 (70.7)	85 (29.3)	0.0142	1.97 (1.11–3.49)*	–	1.77 (0.96–3.25)	1.30 (0.68–2.49)
Depression	3,455 (29.0)	1,980 (57.3)	1,475 (42.7)	0.2397	1.12 (0.93–1.34)	–	–	–
Diabetes without chronic complications	895 (7.5)	460 (51.4)	435 (48.6)	0.2546	0.84 (0.63–1.13)	–	–	–
Diabetes with chronic complications	2,495 (20.9)	1,495 (59.9)	1,000 (40.1)	0.0313	1.26 (1.02–1.57)*	–	1.15 (0.92–1.45)	1.10 (0.87–1.39)
Drug abuse	1,500 (12.6)	980 (65.3)	520 (34.7)	0.0008	1.61 (1.21–2.15)**	–	1.74 (1.30–2.34)***	1.81 (1.34–2.46)***
Hypertension, complicated	2,480 (20.8)	1,535 (61.9)	945 (38.1)	0.0007	1.40 (1.15–1.71)***	–	1.31 (1.06–1.64)*	1.20 (0.95–1.52)
Hypertension, uncomplicated	4,465 (37.4)	2,385 (53.4)	2,080 (46.6)	0.1294	0.88 (0.75–1.04)	–	–	–
Chronic pulmonary disease	4,065 (34.1)	2,240 (55.1)	1,825 (44.9)	0.8403	0.98 (0.83–1.16)	–	–	–
Obesity	2,940 (24.7)	1,690 (57.5)	1,250 (42.5)	0.2373	1.12 (0.93–1.35)	–	–	–
Peripheral vascular disease	1,015 (8.5)	555 (54.7)	460 (45.3)	0.8369	0.97 (0.72–1.30)	–	–	–
Hypothyroidism	2,340 (19.6)	1,295 (55.3)	1,045 (44.7)	0.9827	1.00 (0.81–1.23)	–	–	–
Other thyroid disorders	310 (2.6)	145 (46.8)	165 (53.2)	0.1890	0.70 (0.41–1.19)	–	–	–
Emergency department indicator								
Non-emergency department admission	1,650 (13.8)	950 (57.6)	700 (42.4)	0.4169	Reference	–	–	–
Emergency department admission	10,275 (86.2)	5,655 (55.0)	4,620 (45.0)		0.90 (0.70–1.16)	–	–	–
Major operating room procedure								
No	9,985 (83.7)	5,265 (52.7)	4,720 (47.3)	<.0001	Reference	–	–	Reference
Yes	1,940 (16.3)	1,340 (69.1)	600 (30.9)		2.00 (1.58–2.54)***	–	–	1.68 (1.31–2.15)***
Discharge disposition								
Routine	7,150 (60.0)	3,325 (46.5)	3,825 (53.5)	<.0001	Reference	–	–	Reference
Short-term hospital	260 (2.2)	135 (51.9)	125 (48.1)		1.24 (0.71–2.17)	–	–	1.46 (0.82–2.58)
Other facility^c^	1,775 (14.9)	1,480 (83.4)	295 (16.6)		5.77 (4.29–7.76)***	–	–	5.80 (4.22–7.96)***
Home health care	2,315 (19.4)	1,495 (64.6)	820 (35.4)		2.10 (1.69–2.60)***	–	–	2.16 (1.72–2.71)***
Against medical advice	255 (2.1)	70 (27.5)	185 (72.5)		0.44 (0.23–0.83)*	–	–	0.43 (0.23–0.82)*
Died	170 (1.4)	100 (58.8)	70 (41.2)		1.64 (0.81–3.33)	–	–	1.85 (0.90–3.77)
Hospital region								
Northeast	3,025 (25.4)	1,600 (52.9)	1,425 (47.1)	0.0024	Reference	–	–	Reference
Midwest	2,990 (25.1)	1,685 (56.4)	1,305 (43.6)		1.15 (0.91–1.46)	–	–	1.04 (0.80–1.35)
South	3,675 (30.8)	2,225 (60.5)	1,450 (39.5)		1.37 (1.08–1.73)**	–	–	1.15 (0.88–1.49)
West	2,235 (18.7)	1,095 (49.0)	1,140 (51.0)		0.86 (0.66–1.11)	–	–	0.76 (0.57–1.01)
Hospital bed size^d^								
Small	2,545 (21.3)	1,380 (54.2)	1,165 (45.8)	0.0086	Reference	–	–	–
Medium	3,525 (29.6)	1,795 (50.9)	1,730 (49.1)		0.88 (0.69–1.11)	–	–	–
Large	5,855 (49.1)	3,430 (58.6)	2,425 (41.4)		1.19 (0.95–1.49)	–	–	–
Hospital location/teaching status								
Rural	735 (6.2)	405 (55.1)	330 (44.9)	0.7820	Reference	–	–	–
Urban non-teaching	2,045 (17.1)	1,100 (53.8)	945 (46.2)		0.95 (0.64–1.41)	–	–	–
Urban teaching	9,145 (76.7)	5,100 (55.8)	4,045 (44.2)		1.03 (0.72–1.47)	–	–	–
Hospital control/ownership								
Government, non-federal (public)	975 (8.2)	645 (66.2)	330 (33.8)	0.0122	1.63 (1.05–2.53)*	–	–	1.80 (1.13–2.86)*
Private, not-for-profit (voluntary)	9,950 (83.4)	5,415 (54.4)	4,535 (45.6)		1.00 (0.73–1.37)	–	–	1.04 (0.73–1.48)
Private, investor-owned (proprietary)	1,000 (8.4)	545 (54.5)	455 (45.5)		Reference	–	–	Reference

Abbreviations: AOR, adjusted odds ratio; OR, odds ratio.

^a^Chi-square test.

^b^Includes Asian/Pacific Islander, Native American, two or more races, multiracial, and any race/ethnicity other than White, Black, or Hispanic.

^c^Includes skilled nursing facilities, intermediate care facilities, and other types of facilities.

^d^Bed size is based on a hospital’s total number of beds and is specific to the hospital’s location and teaching status.

***p < 0.001; **p < 0.01; *p < 0.05.

## Data suppressed; 10 or fewer observations in some cells.

### 3.3 Emergency department visits and immediate hospital admission

Descriptive statistics for our analyses of immediate hospitalization are provided in Table 1 along with observed estimates of association. Nearly half (10,868 visits, 47.9% of total) of all CRPS-1 ED visits in 2020 resulted in immediate hospitalization. Bivariable regression models identified a number of characteristics that were independently associated with being admitted to hospital after presenting to the ED with CRPS-1, including but not limited to age, sex, median household income, primary payer, hospital location/teaching status, and hospital trauma level designation. Race/ethnicity was not found to be associated with hospital admission in the bivariable model. Most examined Elixhauser comorbidities were independently associated with hospital admission, with diagnoses of metastatic cancer (odds ratio (OR) 4.91, 95% confidence interval (CI) 2.30 to 10.49) and dementia (OR 4.61, 95% CI 2.72 to 7.80) being most associated with immediate admission.

The addition of covariates to our bivariable models generally resulted in initially observed associations being attenuated in the *fully adjusted model*, though this was not the case for all characteristics. A number of characteristics were found to be more strongly associated with hospitalization in our fully adjusted model. For example, compared with ED visits by White individuals, encounters by Black individuals were significantly negatively associated (adjusted odds ratio (AOR) 0.74, 95% CI 0.55 to 0.99) with immediate hospitalization. Similarly, despite women accounting for approximately three quarters of all CPRS-1 ED visits, ED visits by women were significantly less likely (AOR 0.70, 95% CI 0.58 to 0.85) to result in admission.

The diagnosis of many individual comorbidities remained significantly positively associated with immediate hospitalization after adjustment for potential confounders, with estimates of association being greatest for the diagnosis of acquired immunodeficiency syndrome (AOR 4.90, 95% CI 1.15 to 20.81), drug abuse (AOR 4.67, 95% CI 3.53 to 6.18), and metastatic cancer (AOR 4.36, 95% CI 1.91 to 9.96).

### 3.4 Hospitalizations and longer length of stay

Findings from our analyses of longer length of inpatient stay, including descriptive counts and observed estimates of association, are provided in Table 2. Slightly more than half (6,605 admissions, 55.4% of total) of all examined CRPS-1 hospitalizations were four or more days in length. Characteristics found to be associated with longer length of stay in our bivariable regression analyses included race/ethnicity, median household income, experiencing a major operating room procedure, discharge disposition, hospital region, and hospital control/ownership. Additionally, some individual Elixhauser comorbidities were found to be significantly associated with longer lengths of stay, such as the diagnosis of dementia (OR 1.97, 95% CI 1.11 to 3.49) and drug abuse (OR 1.61, 95% CI 1.21 to 2.15).

A number of originally observed associations were weakened with increasing adjustment for potential confounders in our multivariable models, including those with race/ethnicity, median household income, hospital region, and dementia (Table 2). Nevertheless, many characteristics, such as primary payer, experiencing a major operating room procedure, and discharge disposition, were found to be associated with longer length of stay in the *fully adjusted model*. Compared with CRPS-1 hospitalizations subsidized by private insurance, hospitalizations subsidized by Medicaid showed a significant negative association (AOR 0.68, 95% CI 0.51 to 0.92) with longer length of inpatient stay. Conversely, having a major operating room procedure (AOR 1.68, 95% CI 1.31 to 2.15) and being discharged to another facility (such as a skilled nursing facility relative to routine discharge; AOR 5.80, 95% CI 4.22 to 7.96) were significantly positively associated with increased time in hospital. Drug abuse was the only examined comorbidity to be significantly associated (AOR 1.81, 95% CI 1.34 to 2.46) with longer length of stay in the *fully adjusted model*.

## 4. Discussion

Using nationally representative data from acute care and inpatient settings, we performed cross-sectional analyses with the objectives to increase knowledge of potential race/ethnicity-based disparities in the treatment of complex regional pain syndrome type 1 (CRPS-1). Our primary findings were that, of the more than 23,000 ED visits and over 12,000 hospitalizations related to CRPS-1, most CRPS-1 care was provided to White, female patients. The proportion of females with CRPS-1 across examined race/ethnicity groups was comparable in both clinical settings. Similarly, within all race/ethnicity groups, the number of CRPS-1 ED visits and hospitalizations generally increased with increasing age, often peaking among those aged 50–59 years. Noteworthy secondary findings included: 1) ED visits by Black individuals (compared with White) were significantly negatively associated with immediate hospitalization in the *fully adjusted model*, but not other models; 2) ED visits by females were significantly less likely to result in admission; 3) hospitalizations by Black patients (compared with White) were independently associated with increased length of stay in all models except for the *fully adjusted model* that included all presumed confounders; 4) hospitalizations subsidized by Medicaid (compared with private insurance) showed a significant negative association with longer length of inpatient stay; and 5) the diagnosis of drug abuse was significantly associated with both hospital admission and longer length of stay.

To date, a small number of studies have examined the epidemiology of CRPS, with the incidence rate reported to range from 5.46 (CRPS-1) to 26.2 (CRPS) cases per 100,000 person years [6,19]. The largest population-based study of CRPS-1 in the US was published in 2017 by Elsharydah et al.; it examined diagnoses of CRPS-1 during hospitalizations between 2007 and 2011 using NIS databases. For this study, investigators utilized a series of logistic regression models to determine whether select characteristics were associated with hospitalization where CRPS-1 was diagnosed [7]. Findings revealed racial/ethnicity differences in hospitalization for CRPS-1, whereby the odds of inpatient diagnosis of CRPS-1 was significantly greater during inpatient encounters by White patients as compared with admissions by Black, Hispanic, and Asian patients, respectively. Investigators also reported that there were 22,533 patients with a discharge diagnosis of CRPS-1 in an inpatient sample of 33,406,123 between 2007 and 2011 [7], with other researchers extrapolating these findings without consideration of the source data and reporting the inpatient CRPS-1 incidence proportion to be 0.07% [3,12]. Data analyzed by Elsharydah et al. are encounter-based, not patient-based, and it is not possible to longitudinally follow individual patients within or across multiple years of the NIS [20]. As such, the reported patient numbers actually reflect distinct hospitalizations and not unique patients; the numbers also do not account for repeat hospitalizations by the same patient within or across examined years. Furthermore, the use of survey weighting is notably absent from counts reported by Elsharydah et al., which make it challenging to estimate the actual annual number of adult hospitalizations where CRPS-1 was diagnosed. Our finding that a CRPS-1 diagnosis was documented during 0.04% (40 per 100,000 hospitalizations) of all non-elective adult hospitalizations in 2020 suggests that the inpatient CRPS-1 incidence proportion is considerably lower than previously reported. The decrease in proportion is not presumed to be directly attributed to effects of the COVID-19 pandemic, since total quarterly discharges of hospitalizations where CRPS-1 was recorded were similar across 2020. Additionally, our findings confirm previously reported CRPS-1 epidemiology, including that the majority of encounters/cases are by females and that CRPS-1 often peaks prior to age 60. To our knowledge, our study is the first to thoroughly examine sex and age distributions by race/ethnicity within CRPS-1. Further epidemiological studies are therefore necessary to determine whether similar patterns of acute and inpatient care for CRPS-1 by race/ethnicity are observed across other healthcare settings. Additionally, future research is needed to examine outpatient and primary care in CRPS-1, including enrolment with family physicians, family health teams, and nurse practitioners, as well as utilization of these health services over time. Findings from such studies may serve to inform and thus improve existing and new CRPS-1 diagnostic and treatment strategies.

Our findings provide evidence to suggest that select sociodemographic characteristics, such as race/ethnicity, age, and sex may be associated with hospitalization among adults who presents to the ED with CRPS-1. Notably, in our fully adjusted model, we observed that ED visits by Black individuals (compared with White) and females were significantly negatively associated with immediate hospitalization. These findings complement prior evidence regarding race/ethnicity and sex differences in the diagnosis of CRPS-1, and signal that such disparities may extend beyond initial diagnosis to the subsequent delivery of health services. The observed disparities may be due in-part to numerous factors, including but not limited to differences in pain tolerance and health behaviours [21], as well as implicit bias among healthcare professionals [22,23]. For example, while some studies have demonstrated no evidence of bias in the diagnosis of health conditions, there is growing evidence to suggest that significant differences in care are observed after considering interactions between multiple sociodemographic characteristics (such as race and gender, or race and socioeconomic status) or between sociodemographic characteristics and patient behaviour (such as race and challenging behaviour) [22]. We also observed differences in hospitalization by age in CRPS-1, whereby the odds of admission steadily increased with increasing age. This phenomenon may reflect differences in clinical presentation, comorbidities (including those not associated with the conditions chiefly responsible for precipitating the visit) [24], and care provider practices. Future research should focus on exploring the effects of interactions between sociodemographic characteristics and behaviours on the diagnosis and treatment of CRPS-1.

After comprehensive adjustment for presumed confounders, we found few variables to be associated with length of stay for admissions with a CRPS-1 diagnosis. Race/ethnicity, sex, and age were not associated with length of stay after adjustment, which suggests that inpatient length of stay in CRPS-1 is largely determined by other factors, such as principal diagnosis, comorbidity, and post-operation recovery. Notwithstanding, some characteristics, including primary payer status, revealed associations with length of stay during CRPS-1 hospitalizations. Medicaid beneficiaries were found to have significantly shorter lengths of stay compared with CRPS-1 hospitalizations subsidized by private insurance. Although this contrasts previously reported associations between Medicaid insurance and longer inpatient length of stay (compared with private insurance and uninsured patients, respectively), it may be attributed to the passage of the Affordable Care Act (ACA) and the subsequent expansion of Medicaid eligibility. The expansion of Medicaid eligibility has resulted in many studies examining the effects of insurance status on length of stay, with somewhat inconsistent findings. For example, one study of 2,314 adult inpatients with traumatic injuries found that expansion of Medicaid eligibility (relative to restrictive eligibility) was associated with a significant decrease in length of stay, and that the association between insurance status and length of stay varied by state [25]. A separate study of 4,258,952 general medicine discharges from 211 academic medical centres found that length of stay pre- to post-ACA implementation did not significantly differ between hospitals in Medicaid-expansion compared with non-expansion states [26]. As a result, future epidemiological studies are needed to better understand the impacts of expanded Medicaid eligibility on adult inpatient length of stay, including how expanded coverage may affect care for CRPS-1 by race/ethnicity.

There are numerous strengths to our study. To our knowledge, it is the most comprehensive investigation of CRPS-1 epidemiology to date that leverages nationally representative administrative health data from the US. We report on large, population-based analyses of CRPS-1 using data from both acute care and inpatient settings, which provides valuable insight into CRPS-1 care pathways. Both our selection of CRPS-1 encounters and the identification of Elixhauser comorbidities were based on widely accepted ICD-10 definitions. All of our analyses accounted for the complex sampling design of the NIS and NEDS and utilized sampling weights. This allowed us to compute accurate estimates of ED visits and hospitalizations related to CRPS-1 for calendar year 2020, as well as precise variance estimates for reported associations. Furthermore, due to the availability of rich sociodemographic, clinical, and hospital data within the NIS and NEDS, we were able to assess many distinct exposure-outcome relationships and account for factors suspected of confounding modeled associations. Collectively, our findings provide much needed epidemiological data on ED and inpatient CRPS-1 care by race/ethnicity. They may serve as benchmarks for future studies that examine long-term health outcomes in CRPS-1 or the effectiveness of CRPS-1 interventions.

Some limitations should be considered when interpreting our results. Our reported estimates of ED visits and hospitalizations related to CRPS-1 are based on information documented within administrative records; data from which our estimates are derived are encounter-based and do not contain unique patient identifiers. To our knowledge, there is no validated algorithm to identify CRPS-1 cases within electronic health data and diagnostic criteria for CRPS-1 have generally had poor sensitivity in non-ED settings [27–29]. Moreover, we are unable to account for CRPS-1 diagnoses that are made and solely managed in outpatient clinics. Since CRPS-1 was most often recorded as a secondary diagnosis, it is possible that the diagnosis contributed little to the reason for an ED visit or admission; secondary diagnoses recorded during an ED visit resulting in hospitalization may be from both ED and/or inpatient hospital settings. Collectively, the aforementioned issues introduce the possibility that our reported findings are over or underestimates of actual healthcare encounters related to CRPS-1. As with other studies that utilize administrative health records, it is possible that a subset of our measured exposures and outcomes are misclassified or are not documented at all. Additionally, due to the uniform coding of race/ethnicity within HCUP data, a patient who identifies as both Hispanic and Black would be coded as Hispanic. Such occurrences may result in some of our reported associations being biased. Other study limitations include our inability to account for 1) time lived with CRPS-1; 2) use of prescribed and over-the-counter medications; 3) effects of the COVID-19 pandemic on healthcare availability and behaviours; and 4) our inability to account for mode of ED arrival or perceived acuity in our statistical models. Finally, our study is exploratory in nature, and we therefore did not adjust for multiple comparisons in our statistical models. Notwithstanding these limitations, our study meaningfully adds to the limited literature on CRPS-1 care and may serve as foundation for future health services and outcomes research in this area, including the development and validation of algorithms to identify CRPS-1 cases within electronic health data.

Overall, our study is the largest nationally representative US study to examine ED and inpatient care for CRPS-1, and to report CRPS-1 findings by race/ethnicity. We observed that CRPS-1 care was most often provided to White patients, irrespective of clinical setting, and that race/ethnicity was minimally associated with immediate hospitalization and longer length of stay. Additional studies are required to determine the impact of race/ethnicity on seeking care for CRPS-1, as well the effects of interactions between race/ethnicity and other factors on the diagnosis and treatment of CRPS-1.
